# Attachment styles among students at the faculty of medicine of Sfax and childhood trauma

**DOI:** 10.1192/j.eurpsy.2025.1500

**Published:** 2025-08-26

**Authors:** Y. Yaich, A. Bouallegui, H. Khiari, N. Ben Hamed, A. Hakiri, G. Amri, R. Ghachem

**Affiliations:** 1 Psychiatry B, Razi hospital, Tunis, Tunisia

## Abstract

**Introduction:**

Attachment styles, which describe the patterns of interpersonal relationships formed during early childhood, play a crucial role in shaping individuals’ emotional and social functioning throughout life. Among students at the Faculty of Medicine of Sfax, understanding these attachment styles is particularly relevant, as they can influence academic performance, peer relationships, and overall well-being. Students who experienced adverse childhood events may display heightened anxiety, avoidance, or ambivalence in their relationships, potentially affecting their ability to connect with peers and mentors in the demanding environment of medical education.

**Objectives:**

The purpose of this study was to evaluate the prevalence of different attachment styles among medical externs using the RSQ scale and to examine the relationship between these attachment styles and childhood trauma.

**Methods:**

This is a cross-sectional, descriptive, and analytical study. It was conducted over a period of five months among students at the Faculty of Medicine of Sfax using an online form that included sociodemographic data, medical history, lifestyle habits and the “Relationship Scale Questionnaire” (RSQ).

**Results:**

The average age was 21.63 years. The distribution of students according to their attachment styles showed that avoidant attachment was the most prevalent (29%, n=150), and women exhibited more ambivalent attachment than men (p=0,031). Ambivalent attachment was significantly associated with sexual orientation (p=0,025).

Students with an ambivalent attachment were more likely to be victims of verbal aggression during childhood (p=0.002), whereas students with a disorganized attachment were more likely to be victims of sexual aggression during childhood (p=0.020).This aligns with other studies that have shown that children who have been victims of abuse are more likely to develop insecure attachment, particularly disorganized attachment.

**Conclusions:**

Understanding these dynamics is crucial for developing targeted support systems that promote emotional resilience and healthier relationships within the educational context. The findings suggest that students with insecure attachment styles, often stemming from adverse childhood experiences, may face unique challenges in their academic and social environments. Further research is needed in that area.

**Disclosure of Interest:**

None Declared

